# A Power-Efficient Bio-Potential Acquisition Device with DS-MDE Sensors for Long-Term Healthcare Monitoring Applications

**DOI:** 10.3390/s100504777

**Published:** 2010-05-11

**Authors:** Chia-Lin Chang, Chih-Wei Chang, Hong-Yi Huang, Chen-Ming Hsu, Chia-Hsuan Huang, Jin-Chern Chiou, Ching-Hsing Luo

**Affiliations:** 1 Instrumentation Chip Group, Department of Electric Engineering, National Cheng Kung University, Tainan 701, Taiwan; E-Mails: n2895160@mail.ncku.edu.tw (C.-L.C.); chenming_hsu@hotmail.com (C.-M.H.); sean71123@hotmail.com (C.-H.H.); 2 Department of Electrical Engineering, National Chiao Tung University, Hsinchu, Taiwan; E-Mails: cwchang.ece94g@nctu.edu.tw (C.-W.C.); chiou@mail.nctu.edu.tw (J.-C.C.); 3 Graduate Institute of Electrical Engineering, National Taipei University, Taipei, Taiwan; E-Mail: hyhuang@mail.ntpu.edu.tw; 4 School of Medicine, China Medical University, No. 91, Hsueh-Shih Road, Taichung, Taiwan

**Keywords:** DS-MDE, bio-potential acquisition chip, long-term ECG monitoring

## Abstract

This work describes a power-efficient bio-potential acquisition device for long-term healthcare applications that is implemented using novel microelectromechanical dry electrodes (MDE) and a low power bio-potential processing chip. Using micromachining technology, an attempt is also made to enhance the sensing reliability and stability by fabricating a diamond-shaped MDE (DS-MDE) that has a satisfactory self-stability capability and superior electric conductivity when attached onto skin without any extra skin tissue injury technology. To acquire differential bio-potentials such as ECG signals, the proposed processing chip fabricated in a standard CMOS process has a high common mode rejection ratio (C.M.R.R.) differential amplifier and a 12-bit analog-to-digital converter (ADC). Use of the proposed system and integrate simple peripheral commercial devices can obtain the ECG signal efficiently without additional skin tissue injury and ensure continuous monitoring more than 70 hours with a 400 mAh battery.

## 1. Introduction

Rapid advances in integrated circuit technology have led to the miniaturization of medical monitoring instruments in portable devices for healthcare applications. To satisfy this requirement, an apparatus that simultaneously records bio-potential signals from the surface electrodes on the human body is a priority concern. In addition to recording physiological parameters of patients such as electrocardiogram (ECG), electroencephalogram (EEG) and electromyogram (EMG) signals for diagnosis, such assistant technologies provide convenient access to medical instruments [1–4]. Recording of bio-differential signals such as ECG has also extended into other areas, such as sports medicine and sleep monitoring. A bio-differential signal measurement setup typically consists of the following components [5]:

1. Electrodes at several points on the human body;
2. Analog front end circuits that amplify the bio-differential signals gathered by the electrodes;
3. Analog-to-digital converter (ADC) that digitizes the amplified signals;
4. Processing unit that manages and processes bio-differential signals;
5. Monitoring software that records and displays data.

**Figure 1.**
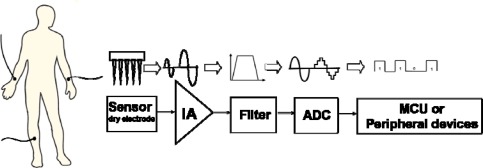
Block diagram of the bio-potential acquisition system.

Figure 1 schematically depicts the proposed bio-potential acquisition system, which includes MEMS-based dry electrodes (MDE), low power bio-potential acquisition circuits and software for monitoring. However, the wet electrodes are uncomfortable for long-term monitoring applications since they always require skin preparation (*i.e.*, abrasion of the outer skin layer) and the use of electrolytic gel to overcome the electrical isolation problem of the outer skin layer. Improper skin preparation might cause skin irritation, pain, or even infections. The use of electrolytic gel is physically uncomfortable and inconvenient; it can cause itchiness, occasionally reddening and skin swelling during long-term ECG-measurement. Furthermore, the conductivity of electrolytic gel decreases gradually due to the hardening of the gel, subsequently degrading the data acquisition quality. Dry electrodes should thus be considered preferable for long-term monitoring systems. Although some dry electrode implementations have low-impedance and excellent signal quality [6,7], the contact problem of these electrodes is still a challenge.

This work presents a diamond-shaped MDE (DS-MDE) to increase the sensing reliability and stability during long-term monitoring. The proposed diamond-like design has a wider neck and a narrower bottom than the general conical shape MDEs. This unique configuration provides an external force to stabilize the probe, so that it can stay in the skin when the tissue counteracts the probe continuously. Additionally, similar to a related work [8] that also demonstrates the mechanical attachment capability of a body surface electrode, the proposed DS-MDE without sharp barded edges does not damage the skin tissue after removing the electrodes from skin. Therefore, the DS-MDE can provide an acceptable self-stability and superior electric conductivity when attached onto skin without causing extra skin tissue injury.

According to Figure 1, the bio-potential acquisition circuits, designed based on 0.18 μm 1P6M CMOS process technology, include an instrumentation amplifier (IA) and an analog-to-digital converter (ADC). Moreover, the common mode noises interfere with the bio-potential signals coupled to a human body. Under these circumstances, front-end circuits are required with the features of high CMRR, low-noise, and filters to extract signals. The proposed processing chip has low power consumption, low noise and high common mode rejection ratio (C.M.R.R.) properties. These features make it feasible for a biomedical signal acquisition system. With the novel MDE sensor and the low power bio-potential acquisition chip, the proposed acquisition device is much more comfortable for human body and more efficient in terms of power for long-term ECG signal acquisition.

## 2. Materials and Methods

### 2.1. DS-MDE

Mobile monitoring requires high-fidelity sensors that transform the bio-potentials from humans to the post-processing circuit and then become integrated with low-power, wireless chipsets. However, current systems [9–11] are limited in terms of large modulator power dissipation, insufficient ECG electrode self-stabilized capability or unrealistic packaging design, severely restricting their applicability in human bio-potential measurements. Consequently, a sensing unit is integrated with MEMS dry electrode (MDE) as a monitoring system (Figure 1). The MDE ECG sensor provides a signal acquisition capability superior to conventional electrodes that must use gel. Moreover, an additional self-stabilized function enhances the sensing reliability and stability during long-term monitoring.

**Figure 2.**
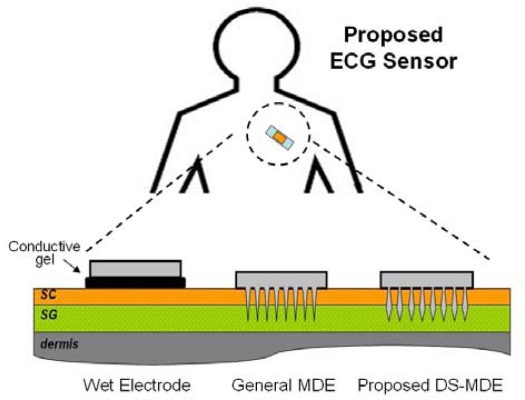
The proposed ECG sensor for bio-signal measurements.

Figure 2 illustrates the proposed ECG sensor for bio-signal measurements. Bio-potential electrodes transform the bio-signals from skin tissue to the processing circuit [12] and produces electrode/skin interface impedance, which acts as a voltage divider with the amplifier’s input resistance for bio-signal transportation. High interface impedance contributes to the thermal noise and signal attenuation to the system [13]. Conventional bio-electrodes, e.g., wet electrodes, must use electric gel to obtain electric conductivity, but this can cause itchiness and irritation during long-term monitoring. Existing designs [14] utilize an electrically conductive microprobe to penetrate the high electrically resistive outer skin layer (stratum corneum, SC), into the deeper skin (stratum germinativum, SG) to obtain electrical conductivity superior to that of wet electrodes [13]. However, the conical shape microprobe array lacks any position stabilization capability. This limitation is due to the force created by the compressed skin tissue that continually counteracts the conical shape microprobes into outward direction and degrades the bio-signal recording quality directly. To overcome this drawback, this work develops a diamond-shaped MDE (DS-MDE). The proposed diamond-like design has a wider neck and a narrower bottom than previous conical shape dry electrodes. This configuration provides an external force to stabilize the probe that can remain in the skin when the tissue counteracts the probe continually. In contrast with a related work [8] which also demonstrates the mechanical attachment capability of a body surface electrode, the proposed DS-MDE without sharp barded edges does not damage the skin tissue after removing the electrodes from skin. The DS-MDE can thus provide satisfying self-stability capability and superior electric conductivity when attached onto skin without additional tissue injury.

**Figure 3.**
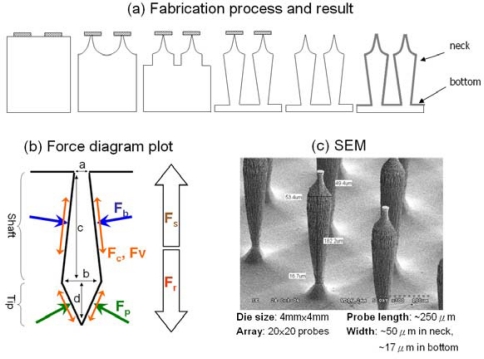
The modeling, fabrication and electrode-skin interface impedance measurement result of the DS-MDE.

Figure 3 summarizes the modeling, fabrication and electrode-skin interface impedance measurement results of the proposed DS-MDE. As shown in the Figure 3(b), the diagram illustrates the forces applied on the probe after the DS-MDE was placed onto skin tissue. ***F****_b_* and ***F****_p_*, caused by the compressed tissue, are the normal force applied on the face of tip and the face of shaft. ***F****_r_* is the resultant force of |***F****_b_* + ***F****_p_*| with direction towards the skin. ***F****_c_* and ***F****_v_* denote the frictional force and viscous force, respectively. ***F****_s_* is the minimal force that required for dragging out the probe from skin tissue. Clearly, the minimal ***F****_s_* for dragging out the probe is:

(1) $$F_{s}\geq F_{r}+\left(F_{c}+F_{v}\right)$$

Since that ***F****_c_* and ***F****_v_* are small, difficult to estimate and always help the probe stay in the tissue when the probes are dragged out of the tissue, they are ignored in the simulation. To quantitatively determine the improved self-stability of the presented DS-MDE comparing with MDE, stability factor (SF) is simply defined as the ***F****_r_*, the resultant force of |***F****_b_* + ***F****_p_*|. It is clear that if ***F****_r_* can provide an inward force into the tissue, the stability of the probe can be improved.

The forces ***F****_b_* and ***F****_p_* are positively proportional to the compressed tissue volume, which can be calculated from the dimension of probe (***a***, ***b***, ***c*** and ***d***) illustrated in the force diagram. Thus, the resultant force ***F****_r_* is calculated from the angle relationship in the force diagram. For the general MDE, its vertical shaft wall (***a*** = ***b***) and conical tip result in a ***F****_r_* with outward direction from the tissue which can not help the probe stay in the skin. Comparing with the MDE, DS-MDE with an invert-triangle shaft wall makes the resultant of ***F****_b_* with the direction towards into the tissue, which helps the probe stay in the skin.

The calculated result indicates that the narrower the bottom width a, the higher the stability that can be achieved is. Note that when the tip length and neck diameter are 50 μm, SF reaches the maximum value if the bottom diameter is less than 30 μm and shaft length is longer than 150 μm. As a result, in this paper a stable probe configuration with 250 μm probe length has been designed to reach the SG layer.

The fabrication process for DS-MDE was developed and illustrated as follows and in Figure 3(a). The DS-MDE microfabrication process consisting of ion etching with inductive coupled plasma (RIE-ICP) etching process and sputtering metallization technology was developed and is illustrated in Figure 3(a). In this process, a 6μm thick photoresist film was patterned as circular hard-mask for the isotropic etching process to produce the probe tip. Next, we proceeded with the anisotropic etching process to form the probe shaft with high aspect ratio. Additionally, the hard mask at the probe tip was released by sulfuric acid wet-etching. Finally, the probes were subsequently coated with titanium and platinum using sputtering technique to achieve electrical conductivity and bio-compatibility.

**Figure 4.**
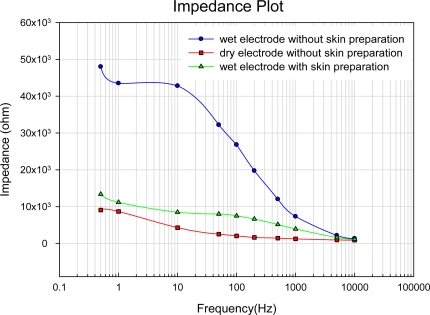
Electrode-skin interface impedance measurement result.

According to Figure 3(c), the fabricated probe is approximately 250 μm in length and 50 μm (neck), 17 μm (bottom) in width. Notably, the peak of the probe was not perfectly sharp (<10 μm) since the tip of the probe must support the hard mask in the second anisotropic etching step. Although not reported herein, a modified process to produce a sharper tip was currently under development. According to Figure 4, the impedance plot indicates that the impedances of a wet electrode with skin preparation (use of gel) and MDE without skin preparation at 10Hz are approximately 9 kΩ and 4 kΩ, respectively.

The self-stabilized capability was tested by gluing a 4 × 4 mm^2^ DS-MDE that contains an array of 20 × 20 micro probes to a PMMA holder for the pulling force test. A pig skin fixed on another PMMA plate was used for the test tissue. The DS-MDE was pressed into the tissue under testing by the holder beam of a micro force testing system (MTS Corp., US). The force with which the DS-MDE was pressed into the tissue was not measured. After ensuring the micro probes penetrated the test tissue, the micro force testing system started to pull off the DS-MDE. During testing, the applied force and displacement were recorded. The average required pulling force for the general MDE and DS-MDE were 1.705 N and 3.160 N, respectively. Notably, both MDE and DS-MDE had the same chip area, number of probes and probe length.

### 2.2. The proposed bio-potential acquisition circuits

**Figure 5.**
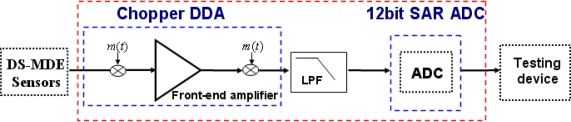
Block diagram of the proposed bio-potential acquisition circuits.

The proposed bio-potential acquisition circuits include front-end amplifiers, filters and ADC, whose block diagram is shown in Figure 5. The front-end amplifiers must possess the following characteristics, high C.M.R.R., high input impedance, low power consumption and low noise for processing biomedical signals. Therefore, the chopper differential difference amplifier (DDA) is adopted as the front-end readout circuit of the system. Following the DDA circuit, a low power filter with low cut-off frequency is selected to suppress the out-of-band noise and provide better signal-to-noise (SNR). However, the acquisition system requires an analog-to-digital converter for digitizing the outputs of the front-end acquisition circuit. A successive approximation register ADC (SAR-ADC) architecture is selected due to its predominant power dissipation property compared to other architectures. In this work, the design of the bio-potential acquisition circuits is fabricated in TSMC 0.18 μm 1P6M CMOS process technology. The acquisition circuits are adopted advanced process in order to integrate radio frequency circuit in next step. The characteristics of the present low power bio-potential acquisition chip could be summarized as follows:

1. High CMRR to reject common mode noise.
2. High input impedance property for DS-MDE sensors.
3. Low noise DDA amplifier for enhancing better signal quality.
4. Sufficient resolution for ECG acquisition.
5. Low power dissipation for long-term monitoring.

#### 2.2.1. The implementation of the proposed bio-potential acquisition chip—DDA

**Figure 6.**
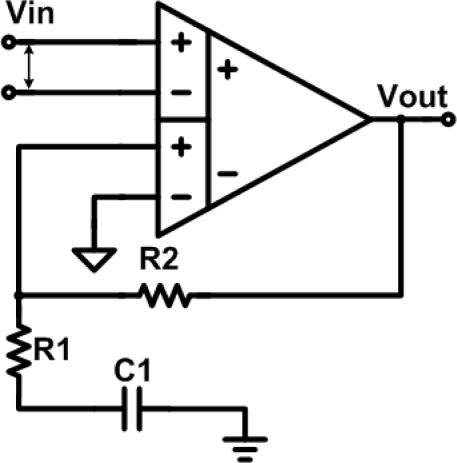
DDA circuit.

Current structures of IA circuits include three op-amp structures [15–18], operational transimpedance amplifier (OTA), current-balance structure (CBIA) [19–22] and DDA [26–28]. The three op-amp IA is a common approach. However, it is very-well known that the CMRR of the three op-amp IA is extremely dependent on the matching of the resistors [23,24]. The resistor matching goal is difficult to achieve in standard CMOS process technology. A second technique is using OTA architectures [25]. Though the OTA could provide proper and stable amplification with negative feedback technique, however, the OTA IA has a poor CMRR performance that just barely meets the minimum specification of 80 dB in ideal simulations. As a result, it doesn’t have enough margin to overcome the process variations. The advantage of CBIA is its simple structure, but on the other hand, it also suffers from the requirement of current-mirror match, which would also affect the CMRR efficiency. In figure 6, the DDA architecture is adopted to achieve high CMRR, high input impedance, low power consumption and low noise performance. These features make it suitable for the proposed bio-potential acquisition system application. The CMRR of DDA only depends on the mismatch of the input pair circuit, not on resistors. The resistors, R1 and R2 only affect the amplification. The DDA transfer function is presented in Equation (1) [27], which reveals that the non-inverting DDA provides unity gain in dc level input signal, and therefore it could suppress the DC offset signal from measuring biomedical signals:

(2) $$\frac{V_{\mathrm{out}}\;(j\omega )}{V_{\mathrm{in}}\;(j\omega )}=\left(\frac{j\omega R_{2}C_{1}}{j\omega R_{1}C_{1}+1}\right)+1$$

The advantage of DDA is its simple structure, which makes DDA circuit possess low power property. In addition to the simple structure, the bias current of the DDA circuit would be adjusted and reduced as small as possible in order to decrease power consumption. But the open loop gain of the DDA circuit must be enough for the specification at the same time.

**Figure 7.**
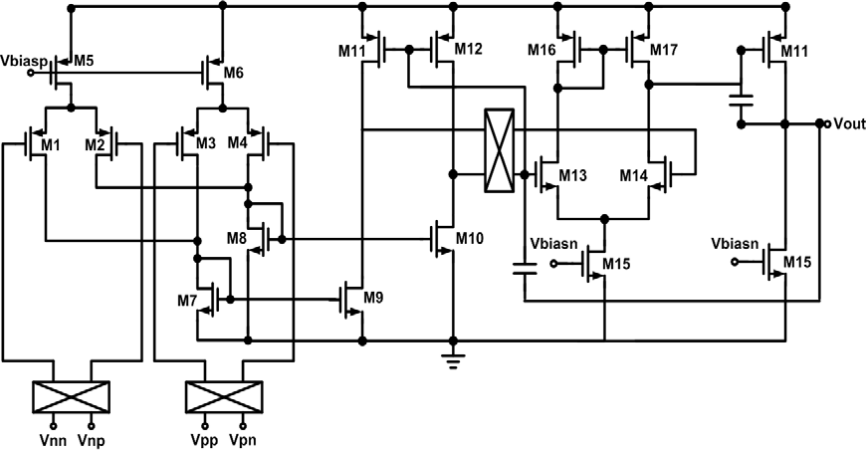
The chopper DDA circuit.

In this work, the front-end amplifier is set to obtain a good balance between power and gain. The DDA circuit is depicted in Figure 7. In order to reduce the original flicker noise, the input pair transistors (M1–M2 and M3–M4) are a longer length PMOS design. The chopper modulation technique [29–31] also could be used for eliminating flicker noise. The input pair transfers the input voltage into current. M9 and M10 construct a common source amplifier. The differential currents flowing through M7 and M8 are transferred back to voltage signal by the load device M11 and M12, which are biased in strong inversion to reduce noise. The output gain stage of DDA is constructed by the two-stage amplifier (M13–M17), which acts as an amplifier. The miller capacitances C are used to increase the phase margin. However, due to the lack of the electrolyte, their characteristics are closer to a polarizable electrode, which can be characterized as a leaky capacitor [27]. Therefore, the readout circuit for DS-MDE electrode must have very high input impedance. The proposed DDA architecture [32] which obtained high input impedance is adopted in the system application. Tabel 1 shows the performance and figure of merit (FOM) of the DDA circuit. The FOM is defined as:

(3) $$\mathrm{FOM}=\left(\frac{\mathrm{CMRR}\times \mathrm{PSRR}}{\mathrm{Power}}\right)$$

#### 2.2.2. Implementation of the proposed bio-potential acquisition chip—ADC

**Figure 8.**
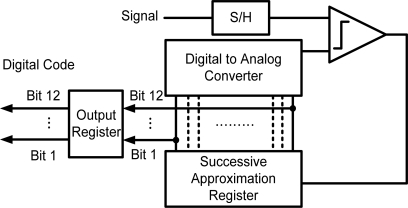
Successive approximation converter architecture.

Portable biomedical instruments must have low power consumption features to enhance the device lifetime for healthcare monitoring applications. The resolution and power consumption lead to an intrinsic trade-off problem [35]. Therefore, in this work, the ADCs in biomedical acquisition systems can not dissipate a significant amount of power. SAR ADC is a conventionally adopted converter for biomedical applications owing to its satisfactory accuracy and low power consumption [33–37]. Figure 8 shows the architecture of a successive approximation A/D converter. The basic converter comprises a sample-and-hold (S/H), comparator, successive approximation register (SAR) and D/A converter. SAR ADC can operate without an amplifier [38,39]. Using a binary search algorithm, the DAC output voltage VDAC successively approximates the sampled input voltage VSH. The binary capacitor array DAC has two features: lower power and large area. In this research, the binary capacitor array DAC is adopted for low power consideration and add a scaling capacitor to reduce the total value of capacitor used in whole DAC. Each clock cycle obtains one bit of the digital output signal.

**Figure 9.**
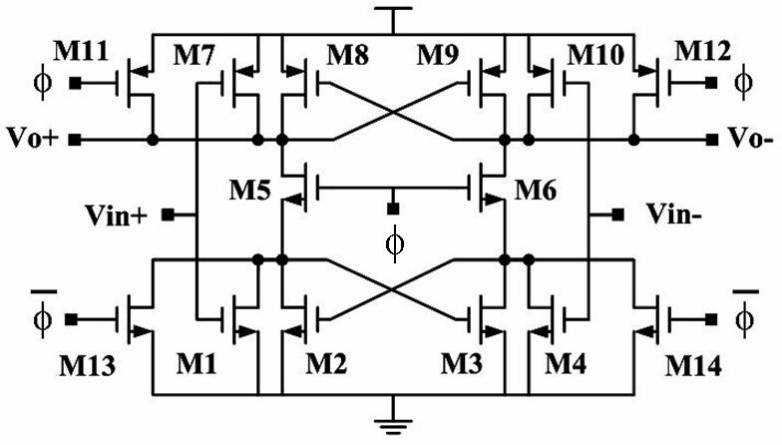
Proposed latch comparator.

A latch comparator is suitable for a low power ADC circuit. This work also develops a novel rail to rail latch comparator (Figure 9) to enlarge the input voltage range and improve the noise tolerance. The power dissipation of the proposed latch comparator is 2.55 μW. The novel rail-to-rail latch comparator is designed for two operating modes, *i.e.*, comparison and sleep mode, for power efficiency.

In the comparator design, PMOS and the NMOS are adopted as the input pair that can provide the rail to rail input range. Regardless of whether input signals are excessively low or high, one pair of PMOS or NMOS still works.

M11, M12, M13, M14 provide a discharge path for the comparator in the sleep (reset) mode. In the comparison mode, the control node ϕ is connected to VDD; in addition, M11, M12, M13, M14 are turned off. In the sleep (reset) mode, ϕ is connected to GND, while M5 M6 are turned off. The DC paths in the comparator are cut off, while M11, M12, M13, M14 provide a path to release the charges stored in the drain and source of M5 and M6 to VDD and GND. Therefore, the outputs (Vo+, Vo−) remain attached to VDD in the sleep (reset) mode.

The comparator offset voltage adds directly to the overall ADC offset. Notably, Vt mismatch in M1, M4, M7 and M10 dominates the offset of a comparator. With the process improvement and advancement, the offset may be lower than 0.5 LSB so that the low power dissipation property can be maintained without using offset cancellation techniques. Notably, a situation in which the offset exceeds 0.5 LSB makes off-chip calibration possible by using the microprocessor [39].

The control signals are generated to operate the system. Sixteen clock cycles (work clock) occur during the hold time, as well as two clock cycles to perform charge redistribution of DAC. The following twelve clock cycles transform a sample signal to a set of digital codes. Finally, the digital codes are stored into the output register after conversion at every other 5-μs at a sampling frequency of 200 kHz.

**Figure 10.**
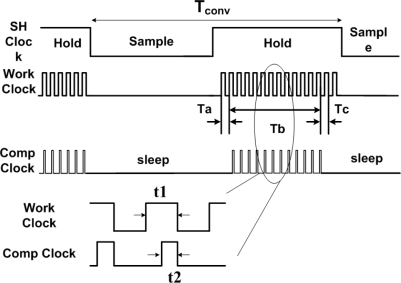
Operation waveforms of the SAR ADC.

The control of signal operation would efficiently affect the power consumption of the SAR ADC [43]. This work proposes an operation method to decrease power dissipation of the SAR ADC circuits and make the operation achieve power efficiency. Figure 10 illustrates the operational waveforms of the signals. Tconv refers to the time involved in converting a voltage to a set of digital codes. In the sample mode, the S/H circuit trace signal, the comparator, SAR and DAC are all in sleep mode, which would reduce power consumption. In the hold mode, S/H circuit stores voltage in the hold capacitor (CH); work clock triggers the DAC. Ta denotes the time of charge redistribution of DAC. After reset of DAC, the work clock triggers SAR and the system begins to convert the holding voltage to a digital code sequentially during the period time (Tb). Following the complete conversion, the digital codes are stored in the output register in the period time (Tc). The comp clock takes control of the comparator in comparison or sleep mode. The comparator is always in sleep mode except operating digital code (Tb). This method makes the comparator efficient, which would result in a great decrease of the total power dissipation. Tabel 2 shows the FOM of the proposed SAR ADC, this index with respect to power consumption, ENOB and samples rate. The FOM is defined as:

(4) $$\mathrm{FOM}=\frac{\mathrm{Power}}{f_{\mathrm{sample}}\cdot 2^{\mathrm{ENOB}}}$$

### 2.3. Peripheral devices for data transmission and storage

The peripheral devices for the proposed system could process and transmit the acquired bio-potential signal to computer for data display and storage. A commercial high-performance, low-power microcontroller could be selected as microcontroller unit (MCU). The MCU could process biomedical analog signals by using the proposed SAR ADC. In the peripheral device, MCU processes and sends the signals via a universal asynchronous receiver/transmitter communication port (UART) or wireless transmission solution [1]. The personal computer could acquire signals through the peripheral devices and process thoroughly by using the software.

## 3. Experimental Results and Discussion

**Figure 11.**
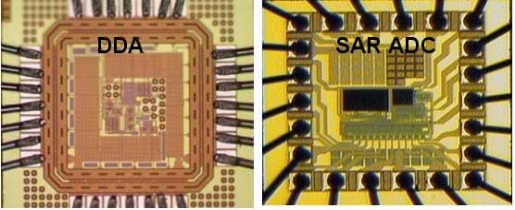
Microphotograph of the fabricated DDA and SAR ADC chip.

Table 3 summarizes the performance of the proposed bio-potential acquisition chip, which includes DDA circuit and SAR ADC circuits. The proposed chip is fabricated (Figure 11) in by a 0.18 μm CMOS process. This work presents a low power bio-potential acquisition chip that is feasible for energy-constrained portable monitoring devices. Moreover, the front-end DDA has achieved a high C.M.R.R., high input impedance, low power consumption and low noise, thus satisfying requirements for ECG signals acquisition. The measured effective number of bits (ENOB) of the SAR ADC is 9.4 bits, which is still higher than the required resolution of processing ECG signals (7∼8 bits) [40]. Therefore, in addition to providing sufficient resolution, the proposed SAR ADC also achieves low power consumption.

**Figure 12.**
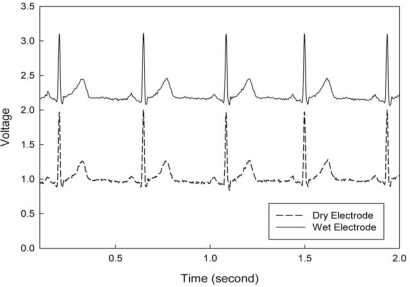
ECG signals sensed by the proposed bio-potential acquisition circuits.

**Figure 13.**
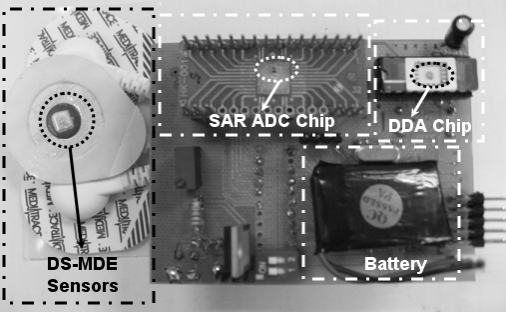
Picture of the proposed acquisition device.

Figure 13 presents the proposed device, including DS-MDE and low power bio-potential acquisition chips. Area of the tested PCB is 5.5 × 7.5 cm^2^, and the device can be powered by batteries. Long-term monitoring is achieved using the proposed DS-MDE for signal acquisition. Figure 12 indicates that the proposed bio-potential acquisition system clearly monitors the PQRST information of lead II ECG signals: significant features of the waveform are the P, Q, R, S, and T waves, the duration of each wave, and particular time intervals. Moreover, the P, Q, R, S and T functions can be used for ECG monitoring and analysis.

Table 4 summarizes the power consumption of the two modes for the proposed acquisition device integrated with commercial peripheral devices. In the cable mode, the peripheral device includes MCU and UART communication chip (MAX3232). The MCU could obtain biomedical analog signals by using the proposed processing chip and transmits the signals via UART port to PC. Additionally, this system can operate about 73 hours with a 400 mAh battery. In the wireless mode, the device contains MCU and wireless ZigBee module. The MCU can transmit the biomedical signals with wireless solution, and the device lifetime about 25 hours. A wireless solution is more feasible for portable monitoring applications even though it consumes more power.

According to the measurement data, the power consumption of the proposed processing chip is about 328 uW, *i.e.*, significantly smaller than the peripheral device (17.4 mW and 37.3 mW), and can be individually operated for about 2 months with a 400 mAh battery. With these low power and high performing characteristics, the proposed device can be integrated with other low power devices for various long-term healthcare monitoring applications. For instance, the proposed device can be integrated with flash memory for long-term data storage, or a cell phone module for normal life monitoring applications.

## 4. Conclusions

The proposed device, implemented by DS-MDE sensors, low power bio-potential acquisition circuits and software for monitoring, was successively proven to acquire ECG signals. This device not only improves the comfort of wearing sensors, but also can be integrated with different devices for long-term applications. The measurement results shows the miniaturized biomedical sensing device can be either worn or implanted by the patients and it would be led into more individualized health care services in the future.

## Figures and Tables

**Table 1.** Measured performance summary of the DDA circuit.

	**This work**	**IEEE TCAS-I [27]**	**IEEE TIM [22]**
IA type	DDA	DDA	CBIA
CMRR[dB]	83	120	99
PSRR[dB]	67	52	40
Power[μW]	277	1455	292
Process[μm] CMOS	0.18	0.5	2.4
FOM	20.07	4.29	16.56
External components	R and C	R and C	R and C

**Table 2.** Measured performance summary of the SAR ADC chip.

	**This work**	**IEEE JSSC[42]**	**IEEE JSSC[41]**	**ISCAS [37]**
Supply voltage[V]	1.8	1	5	3.3
Process[μm] CMOS	0.18	1.2 (SOS)	3	3.3
Resolution[bits]	12	8	8	8
Sample Rate [KHz]	200	50	1300	1230
Input Range[V]	1.8	0.85	3	2.11
ENOB@10KHz[bits]	9.4	7.9	7.85	7.92
Power[uW]	50.58	340	70000	1500
FOM[pJ/conv.step]	0.37	28.4	233	5.11

**Table 3.** Performance summary of the bio-potential acquisition device.

**DDA circuit**	Mid-band gain(dB)	40 to 78
	PSRR(dB)	67
	CMRR(dB)	83
	Power dissipation (uW)	277

**SAR ADC**	Resolution (Bit)	12
	ENOB (Bits)	9.4
	Power dissipation (uW)	50.58

**Table 4.** Two modes’ power consumption of the proposed acquisition device integrated with commercial peripheral devices.

Cable mode	The processing chip	MCU	MAX3232
Power dissipation (mW)	0.328	14.1	3.3
Power dissipation (%)	1.9	79.5	18.6
Device lifetime	73 hours		

Wireless mode	The processing chip	MCU	ZigBee module
Power dissipation (mW)	0.328	14.1	23.2
Power dissipation (%)	0.87	37.47	61.66
Device lifetime	25 hours		
